# Draft Genome Sequence of *Flavihumibacter* sp. Strain CACIAM 22H1, a Heterotrophic Bacterium Associated with Cyanobacteria

**DOI:** 10.1128/genomeA.00400-16

**Published:** 2016-05-19

**Authors:** Pablo Henrique Gonçalves Moraes, Alex Ranieri Jerônimo Lima, Andrei Santos Siqueira, Leonardo Teixeira Dall’Agnol, Anna Rafaella Ferreira Baraúna, Délia Cristina Figueira Aguiar, Hellen Thais Fuzii, Keila Cristina Ferreira Albuquerque, Clayton Pereira Silva de Lima, Márcio Roberto Teixeira Nunes, João Lídio Silva Gonçalves Vianez-Júnior, Evonnildo Costa Gonçalves

**Affiliations:** a Laboratório de Tecnologia Biomolecular, Instituto de Ciências Biológicas (ICB), Universidade Federal do Pará (UFPA), Belém, Pará, Brazil; b Universidade Federal do Maranhão (UFMA), Campus de Bacabal, Maranhão, Brazil; c Laboratório de Patologia Clínica e Doenças Tropicais, Núcleo de Medicina Tropical, Universidade Federal do Pará, Belém, Pará, Brazil; d Centro de Inovações Tecnológicas (CIT), Instituto Evandro Chagas (IEC), Ananindeua, Pará, Brazil

## Abstract

Here, we present a draft genome and annotation of *Flavihumibacter* sp. CACIAM 22H1, isolated from Bolonha Lake, Brazil, which will provide further insight into the production of substances of biotechnological interest.

## GENOME ANNOUNCEMENT

The genus *Flavihumibacter*, as previously described (1), belongs to the phylum *Bacteroidetes* and includes three recognized species: *Flavihumibacter petaseus* T41^T^ (1), *Flavihumibacter cheonanensis* WS16^T^ (2), and *Flavihumibacter solisilvae* 3-3^T^ (3).

The draft genome sequence obtained for the heterotrophic bacterium *Flavihumibacter* sp. strain CACIAM 22H1 was recovered from total DNA obtained from a nonaxenic culture of the cyanobacterium *Tolypothrix* sp. strain CACIAM 22, which was isolated from a water sample from Bolonha Lake (1°25′00.7″S, 48°25′52.6″W), Belém, Pará, Brazil.

After DNA extraction of the cyanobacterial culture, two sequencing runs were performed by GS FLX 454 sequencer (Roche Life Sciences) using a nonpaired library and one sequencing run was carried out on the Illumina MiSeq platform with a paired-end library with 150-bp read length. The raw reads generated after three sequencing runs were processed, resulting in 3,113,618 quality-filtered trimmed reads, followed by coassembly of all datasets using gsAssembler software (Newbler v2.9) with the following parameters: minimum overlap of 20 bp, minimum overlap identity of 80%, heterozygote mode, and extend low-depth overlap options on.

MaxBin 2.0 (4), which employs an expectation-maximization algorithm, was used to bin the assembled sequences. To taxonomically classify the obtained bins, we performed BLASTp for each bin in the sequences containing hidden Markov models for essential genes identified by MaxBin 2.0 against the NCBI nonredundant database. The results were visualized on MEGAN 5.11.3 (5).

Genome structural annotation was carried out using the NCBI Prokaryotic Genome Annotation Pipeline (6). The genome size is 4,877,258 bp, with a mean G+C content of 43.86%, 170 scaffolds, and a *N*_50_ value of 46,868. The genome includes 35 tRNA genes, 2 rRNA genes, 483 pseudo genes, 3 noncoding RNA genes, and 4,271 protein-coding sequences.

Our draft genome of *Flavihumibacter* sp. CACIAM 22H1 possesses 103 (including duplication of Ribosomal L6 and TIGR00234) of the 107 essential genes used as models by MaxBin 2.0, allowing completeness near 94.4%. Overall, the data presented here should improve the genomic information about this poorly studied genus *Flavihumibacter* as well as its association with cyanobacteria. Furthermore, it should provide further insight into the production of substances of biotechnological interest.

### Nucleotide sequence accession numbers.

This whole-genome shotgun project has been deposited at DDBJ/EMBL/GenBank under the acession no. LUKG00000000. The version described in this paper is version LUKG01000000.
